# Pharmacological activation of epidermal growth factor receptor signaling inhibits colitis-associated cancer in mice

**DOI:** 10.1038/s41598-018-27353-w

**Published:** 2018-06-14

**Authors:** Philip E. Dubé, Cambrian Y. Liu, Nandini Girish, M. Kay Washington, D. Brent Polk

**Affiliations:** 1 Division of Pediatric Gastroenterology, Hepatology, and Nutrition, Children’s Hospital Los Angeles, Los Angeles, CA USA; 2 Department of Pathology, Vanderbilt University Medical Center, Nashville, TN USA; 3 Department of Biochemistry and Molecular Medicine, Keck School of Medicine of University of Southern California, Los Angeles, CA USA; 4 Present Address: Taconic Biosciences, Hudson, NY USA

## Abstract

Current treatments for inflammatory bowel disease (IBD) target the overactive immune response of the intestinal mucosa. However, epidermal growth factor (EGF), an activating ligand of the EGF receptor (EGFR), has been shown to induce disease remission through direct targeting of intestinal mucosal healing. Despite promising preclinical and clinical results, this EGFR-activating therapy has not progressed, in part due to the potential for carcinogenesis associated with long-term use and the increased risk of colitis-associated cancer (CAC) in IBD. Here we tested whether pharmacological modulation of EGFR altered outcomes of CAC in the murine azoxymethane/dextran sulfate sodium model. We found that administering EGF during the period of maximum colitis severity (“early”), coincident with the initiation and early promotion of tumors, improved outcomes of colitis and reduced tumor size. In contrast, daily EGF administration beginning ~2 months after tumor initiation (“late”) increased tumor size. Administration of the EGFR kinase inhibitor gefitinib increased the tumor size when the drug was given early and decreased the tumor size when the drug was administered late. EGF administration not only reduced colonic cytokine and chemokine expression during injury, but also baseline chemokine expression in homeostasis. These results suggest that EGFR activation during acute bouts of colitis may reduce the long-term burden of CAC.

## Introduction

Inflammatory bowel disease (IBD) affects ~1.5 million Americans and millions more globally^1–3^. Current and emerging treatments for IBD primarily target the overactive immune response, which can cycle through states of relapse and remission. Inhibitors of tumor necrosis factor^4^, JAK-STAT signaling^5^, interleukin-12/23^6–8^, and integrin pathways involved in leukocyte extravasation^9^ have demonstrated first and second-line effectiveness in patients with Crohn’s disease and ulcerative colitis (UC). Typical clinical response rates for emerging immune-targeted therapies are between 40–70%, with ~40% of patients achieving remission. However, therapeutic effects are eventually lost in 10–50% of primary responders. These data support the need for improved therapies targeting distinct pathways involved in disease pathogenesis^10–18^.

Intestinal mucosal healing is a marker of remission and a predictor of long-term positive outcomes in IBD^19–23^. However, none of the currently approved medications for IBD directly target this process, and in fact some may inhibit intestinal epithelial wound repair^24–29^. Given the important barrier role the intestinal epithelium plays in regulating host exposure to the microbiome and to other luminal contents, and the discovery of genetic factors linked to defective epithelial restitution in IBD^30–32^, the wound healing process has emerged as an attractive therapeutic target. Epithelial repair is mediated by numerous autocrine and paracrine signals, including growth factors and cytokines^33,34^. Epidermal growth factor (EGF) receptor (R) activity is critical for efficient intestinal epithelial wound healing. EGFR has seven known ligands (EGF, TGFα, HB-EGF, betacellulin, amphiregulin, epiregulin, and epigen). EGF is the most well-characterized ligand of EGFR and is expressed in Brunner’s glands of the duodenum and in Paneth cells in the lower gastrointestinal tract of adult rodents^35,36^. TGFα is abundantly expressed in uninjured intestinal crypts and villi and is thought to be the predominant EGFR ligand in the gastrointestinal tract^35,37^. Amphiregulin and epiregulin are expressed at low levels in human colonic mucosa, but demonstrate upregulated expression in IBD and prevent mucosal damage in rodent colitis^38,39^. EGFR activation by its ligands promotes intestinal epithelial cell migration, proliferation, and survival^40–42^. Complementing local production in the intestine, EGFR ligands are found in the intestinal lumen and can cross the compromised epithelial barrier to activate basolateral epithelial EGFR molecules specifically in times of injury^43–47^. In addition, in preclinical models of colonic injury, the expression of EGFR ligands increases^39,48–50^. Loss of EGFR signaling in mice results in impaired restitution and worsened outcomes of colitis^51,52^. In human IBD, the *ERRFI1* gene encoding for a feedback inhibitor of EGFR signaling has been identified as a potential risk locus^53,54^; thus a subset of patients may exhibit abnormal EGFR signaling and may benefit from new therapies targeting this pathway.

A small, short-term clinical trial demonstrated the effectiveness of EGF enemas in the treatment of patients with UC; more than 80% of patients achieved clinical remission^55^. Despite these encouraging clinical results, EGFR-activating therapies for IBD have not advanced. There is a theoretical risk of carcinogenesis associated with longer-term EGFR-activating therapy. EGFR mutations, gene amplifications, and overexpression are central features in a variety of human malignancies, including colorectal cancer, and biologic EGFR inhibitors such as cetuximab and panitumumab are commonly used for their anti-oncogenic activity^56,57^.

Preclinical studies using mice harboring dominant-negative EGFR kinase mutations have shown, paradoxically, increased carcinogenesis in the context of IBD^52^. These effects may vary depending on animal facility, mouse strain, and microbiome status^58^. In contrast, EGFR activation promotes the growth and aggressiveness of modeled sporadic/familial intestinal tumors in mice with genetic disruptions in *Apc*^59,60^. EGFR activation may therefore act specifically in the inflammatory tumorigenic process to suppress colitis-associated cancer (CAC), a serious consequence of long-term intestinal inflammation^61,62^. The mechanism of how this tumor-suppressive role of EGFR works is not known. Moreover, it is unclear whether EGFR modulation using a pharmacologically relevant approach would yield similar results with respect to CAC burden. Here, we have examined these issues using an animal model of colitis and CAC that allows testing the effects of EGFR modulation at different timepoints emulating active flares, healing, and remission in IBD patients. We report that while EGFR activation during injury reduces overall tumor burden, receptor activation long after mucosal healing and tumor initiation increases tumor burden. These results highlight the potential consequences in administering EGFR-directed therapy during different states of intestinal disease. and suggest that the use of this therapy during active flares may lead to better outcomes.

## Materials and Methods

### Mice

Mice were maintained humanely and ethically, in accordance with regulations of the Institutional Animal Care and Use Committee (IACUC) at Children’s Hospital Los Angeles (CHLA). This study was approved by the CHLA IACUC under the internal protocol number 288. All experiments were performed on C57Bl/6 J mice ordered from Jackson Laboratories (stock #000664). Mice arrived at CHLA at 6 wks of age and were co-housed for 6 wks prior to the commencement of experimentation.

### Azoxymethane/dextran sulfate sodium colitis-associated cancer model

Azoxymethane (AOM) was purchased from Sigma (cat. #A5486) and diluted in water to a stock concentration of 2.5 mg/ml. AOM was injected intraperitoneally in a 0.1 ml volume to a target dose of 12.5 mg/kg. Dextran sulfate sodium (DSS, ~40 kDa) was purchased from Affymetrix (cat #14489) and diluted to 3% w/v in sterile water. The DSS solution was provided for 6 d as the sole water source to mice that had been pre-conditioned for >1 wk to accept a drinking bottle source.

### Pharmacological treatments

Gefitinib was purchased from LC Laboratories (cat #G-4408) and diluted for oral gavage in Tween-80 (1% v/v), carboxymethylcellulose (0.1% w/v), and sucrose (1% w/v) to a working concentration of 40 mg/ml. Mice were gavaged daily with a volume of 0.1 ml (4 mg dose, equivalent to ~200 mg/kg). Murine EGF was purchased from Peprotech (cat #315-09) and diluted to a working concentration of 5 µg/ml in sterile 0.9% NaCl for injection. A daily volume of 0.2 ml (1 µg) was used intraperitoneal injections.

### Histology

Mice were anesthetized with isoflurane and euthanized via cervical dislocation. The colon was removed from the abdominal cavity, opened longitudinally, and washed of feces. Colons were weighed, and their lengths were measured. With the exception of a small piece used for RNA isolation and gene expression analysis, the colon was flattened and fixed in 10% neutral buffered formalin at room temperature overnight. Colons were washed with water and dehydrated through an ascending ethanol series. Paraffin embedding, sectioning, and staining with hematoxylin and eosin (H&E) were performed according to standard protocols.

For immunohistochemistry, unstained sections of 5 µm thickness from colonic samples were deparaffinized and boiled in 10 mM citrate/0.05% Tween-20, pH 6.0 buffer. Endogenous peroxidases were bleached with a 3% hydrogen peroxide/PBS solution for 30 min at room temperature. Primary antibodies were rat anti-KI-67 (Dako, cat. #M7249, 1:200 dilution, and eBioscience, cat. #41-5698 & clone SolA15, 1:300 dilution) and rabbit anti-CTNNB1 (Cell Signaling Technology, cat. #9582, 1:300 dilution). EnVision HRP-conjugated secondary antibodies (Dako) were used with DAB pellets (Sigma) to develop the stain. Tissue slides were counterstained with hematoxylin, blued with ammonium hydroxide, dehydated, treated with xylenes, and mounted with Permount (Electron Microscopy Sciences). To quantify KI-67+ (proliferating) cells, the number of labeled nuclei were counted from 25 distal colonic crypts and divided to obtain an average per-crypt proliferative index for each animal. Measurements of crypt depth were obtained by measuring the pixel distance from base to luminal surface in 5 orthogonally sliced distal colonic crypts per animal in ImageJ. The average crypt depth per animal was then compared across all animals in the control and experimental groups.

### Colitis scoring

Histologic assessment of colitis severity was performed in a blinded manner from H&E-stained sections by a mouse pathologist (MKW), similarly as previously described^63,64^. The scoring system ranged from 0–20, with 20 indicating the worst injury. Total scores were computed by summing individual scores (0–4) across five categories of histological features associated with DSS-induced colonic mucosal pathology: the amount of inflammatory infiltrate, depth of inflammation, fraction of crypts involved by the inflammation, crypt damage, and the fraction of crypts involved by crypt damage.

### Gene expression analysis

A 1-mm longitudinal sliver of freshly dissected mouse colon was homogenized with tungsten carbide beads (Qiagen) in a Qiagen TissueLyser LT homogenizer. RNA was isolated using the Ambion PureLink mini-kit. cDNA was prepared using the Bio-rad iScript reverse transcriptase. Quantitative PCR was performed using reagents from the Maxima qPCR kit with ROX background dye on a Bio-rad iQ5 camera-equipped thermocycler for 40 cycles. Primer and probe sequences for the analyzed genes are as follows: *Cxcl2*: CATCCAGAGCTTGAGTGTGA (forward), CCCTTGAGAGTGGCTATGACTT (reverse), CTGCGCCCCAGACAGAAGT (probe); *Il6*: CTTCACAAGTCGGAGGCTTA (forward), GAATTGCCATTGCACAACTCT (reverse), TCGTGGAAATGAGAAAG (probe); *Ifng*: TGCCAAGTTTGAGGTCAAC (forward), GAATCAGCAGCGACTCCTTT (reverse), CTCAGGAAGCGGAAAAG (probe); *Il17a*: TGGACTCTCCACCGCAATG (forward), TCAGGACCAGGATCTCTTG (reverse), TGTTCTCATCCAGCAAG (probe).

C_T_ values were extracted using the log-line method and compared with a reference gene (*Actb*) to derive ΔC_T_ values. ΔΔC_T_ values for cytokines involved in DSS colitis were obtained by comparing the respective ΔC_T_ values to those of uninjured (water-treated) specimens. Statistical tests were performed on the Gaussian-distributed ΔΔC_T_ values, which were subsequently transformed to obtain fold-change estimates.

### Statistics

Statistical tests were performed in Prism 7 (Graphpad). The t-test was performed assuming equal variances between conditions. The cutoff for significance was set at p < 0.05, unless otherwise noted.

## Results

### Effects of EGFR inhibition in injury and inflammation

**Figure 1:**
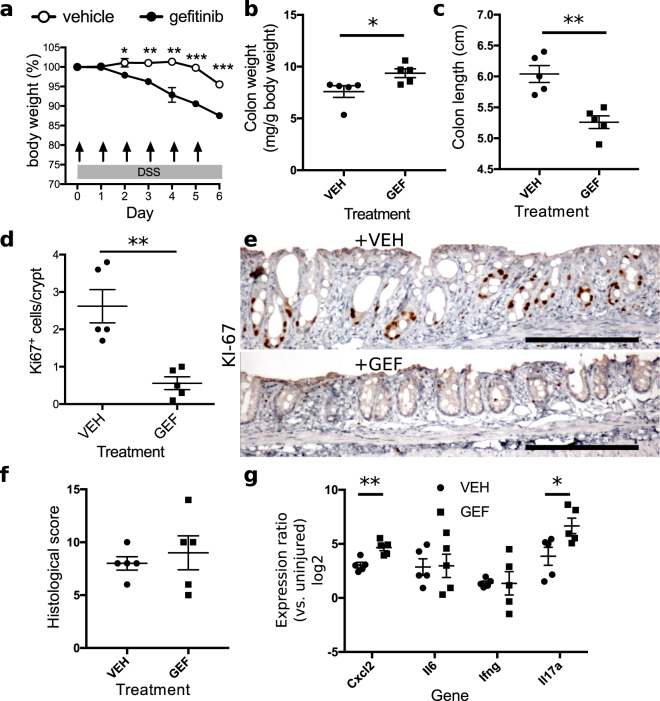
Inhibition of EGFR kinase activity exacerbates colonic mucosal injury. (**a**) Concomitant gavage of mice with 200 mg/kg/d gefitinib and 3% DSS administration leads to accelerated weight loss, which was apparent by day 2, in gefitinib-treated animals compared to vehicle-treated controls. Arrows denote days in which a single daily dose of vehicle or gefitinib was administered (n = 5 mice per group). (**b**,**c**) Gefitinib (GEF) treatment increased colonic weight (**b**) and decreased colonic length (**c**), compared to vehicle (VEH) treatment. These changes correlate with increased injury. (**d**,**e**) Gefitinib (GEF) reduced the number (**d**) of proliferating, KI-67+ epithelial cells, as illustrated in representative stainings (**e**) comparing vehicle (VEH)- and gefitinib (GEF)-treated samples (stained KI-67 is brown with counterstained blue hematoxylin). Images were acquired at 100× magnification. (**f**) No differences between samples were observed in histological score at day 6. (**g**) Gefitinib treatment elevated colonic mRNA expression levels of *Cxcl2* and *Il17a* cytokines (day 6). *p < 0.05; **p < 0.01; ***p < 0.001. Summary statistics: mean ± s.e.m. Scale bars: 200 µm.

Our prior findings showed genetic disruption of EGFR caused earlier onset and more-severe colitis in susceptible (*i.e., Il10*^*−/−*^) mice^52^. To test whether pharmacological inhibition of EGFR kinase activity increased colonic injury and inflammation in wildtype adult mice, we orally administered 200 mg/kg gefitinib to mice exposed to the dextran sulfate sodium (DSS) colitis model. To assess EGFR’s effects specifically in preventing the onset of injury, mice were given 3% DSS through their drinking water for 6 d and euthanized at the end of the treatment. Gefitinib or control diluent (vehicle) were gavaged on day 0 through day 5 (i.e., during the DSS treatment). Mice given the vehicle control exhibited minimal weight loss (<5% of initial body weight), but those given gefitinib lost >10% of their initial body weight by day 6 (n = 5 mice per condition, Fig. 1a). The postmortem colons of gefitinib-treated animals were 31% heavier (p = 0.03, Fig. 1b) and 13% shorter (p = 0.002, Fig. 1c) than the colons of controls, consistent with increased injury. Treatment with gefitinib was associated with a reduction of 79% (p = 0.003) in the number of KI-67+ (mitotic) epithelial cells in colonic crypts (Fig. 1d,e), supporting the relative lack of epithelial proliferative response to inflammation. The histological appearance of the DSS colitis was blind-scored and revealed moderate colitis (median score: 10 of a maximum of 20), but the scores between gefitinib- and vehicle-treated groups did not differ (p = 0.99, Fig. 1f).

To characterize the effects of gefitinib on DSS-associated inflammation, we examined the expression levels of four chemokines/cytokines (*Cxcl2*, *Il6*, *Ifng*, *Il17a*) that reflect fundamental inflammatory processes in DSS-induced colonic injury. *Cxcl2* is a chemoattractant for neutrophils and is secreted by macrophages^65^; these cells represent two of the primary immune cell types infiltrating the colonic mucosa in DSS colitis^66^, and mice deficient in CXCR2, a CXCL2 receptor, are protected from DSS colitis^67,68^. Likewise, the ablation of IL-6, an acute phase cytokine responsible for initiating broad immune responses, has also been linked to improved outcomes of DSS^69,70^. Cytokine profiling of acute DSS injury has demonstrated a skewed Th1/17 profile^71^; thus, we analyzed expression levels of representative cytokines of Th1 (IFNγ)^72,73^ and Th17 (IL-17) inflammation^74–76^ that have demonstrated fundamental roles in pathogenesis. Real-time quantitative PCR analysis of *Cxcl2*, *Il6*, *Ifng*, and *Il17a* demonstrated increased levels of *Cxcl2* and *Il17a*, but not *Il6* (p = 0.94) or *Ifng* (p = 0.98), in gefitinib-treated colons (Fig. 1g). For example, colonic *Cxcl2* was elevated 8.2 times in DSS-injured animals versus uninjured animals; treatment with gefitinib increased *Cxcl2* expression to a total upregulation of 25 times over uninjured animals (p = 0.003). Likewise, colonic *Il17a* was increased 14 times in DSS-injured animals compared to uninjured animals, and gefinitib further increased this upregulation to 101 times the uninjured baseline expression value (p = 0.03).

**Figure 2:**
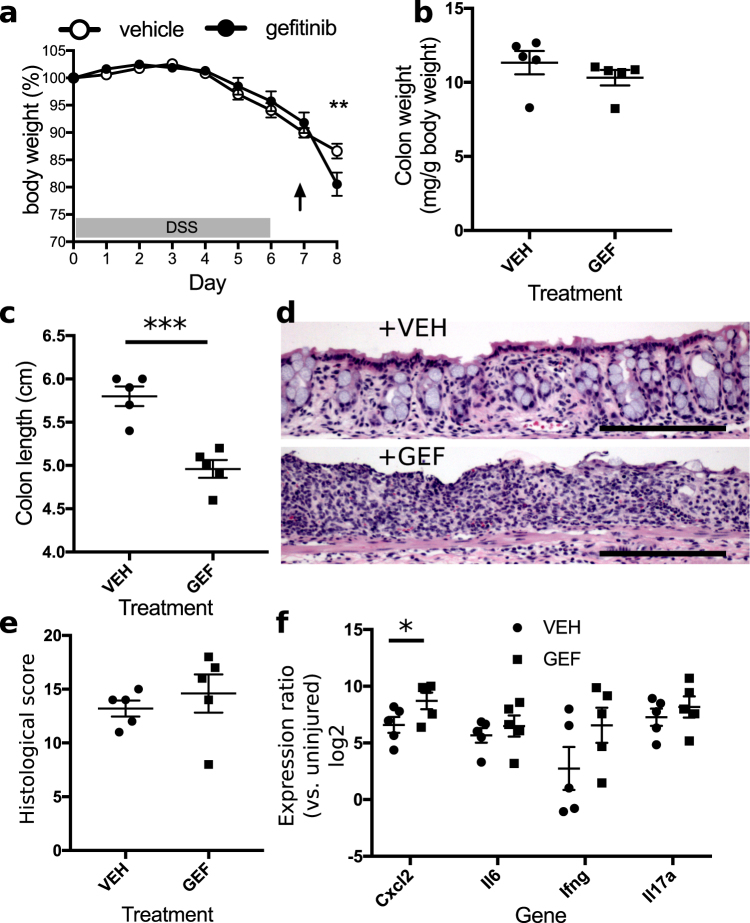
EGFR inhibition impairs mucosal recovery from colitis. (**a**) Administration of 200 mg/kg gefitinib after completion of a 6-day-treatment of 3% DSS resulted in severe body weight loss. The arrow denotes the day in which a single dose of vehicle or gefitinib was administered (n = 5 mice per group). (**b**,**c**) Colonic weights on day 8 were unchanged between mice treated with gefitinib (GEF) or vehicle (VEH) (**b**), but colonic length was reduced in gefitinib-treated samples (**c**). (**d**) Representative hematoxylin and eosin (H&E)-stained sections of injured colon demonstrated increased crypt loss associated with gefinitib (GEF) treatment versus vehicle (VEH). Images were acquired at 40× magnification. (**e**) There was no change in histological score between groups. (**f**) *Cxcl2* expression increased in gefitinib (GEF)-treated samples. *p < 0.05; **p < 0.01; ***p < 0.001. Summary statistics: mean ± s.e.m. Scale bars: 200 µm.

Colonic mucosal healing begins with the cessation of DSS treatment. In a separate group of mice, we had planned to examine the effects of EGFR kinase inhibition during the healing phase by administering gefitinib daily for >2 d beginning with the withdrawal of DSS. However, during the experiment we found that only a single dose of gefitinib was needed to observe a difference in outcomes. While vehicle-adminstered animals continued a pattern of gradual body weight loss, gefitinib-treated animals exhibited a precipitous decline in body weight. This decline necessitated euthanasia of gefitinib-treated animals for humane reasons and discontinuation of the experiment. We also euthanized vehicle-administered animals on this day to enable proper postmortem analysis and comparisons (n = 5 mice per condition, Fig. 2a). Colonic weights did not differ between groups (p = 0.32, Fig. 2b), but colonic length was 14% shorter in gefitinib-treated animals (Fig. 2c, p = 0.002), consistent with fast tissue contraction in response to increased injury^77^. On H&E-stained sections (Fig. 2d), colons from gefitinib-treated animals exhibited regions with complete loss of epithelial architecture, but colons from vehicle-treated animals still harbored remnants of crypts. Histological scores of colitis were not different between groups (p = 0.49, Fig. 2e). RNA expression levels of *Cxcl2* were significantly increased by 4.3 times (p = 0.04), but levels of *Il6* (p = 0.49), *Ifng* (p = 0.16), and *Il17a* (p = 0.47) were not significantly changed (Fig. 2f).

### Effects of EGFR activation in DSS colitis

**Figure 3:**
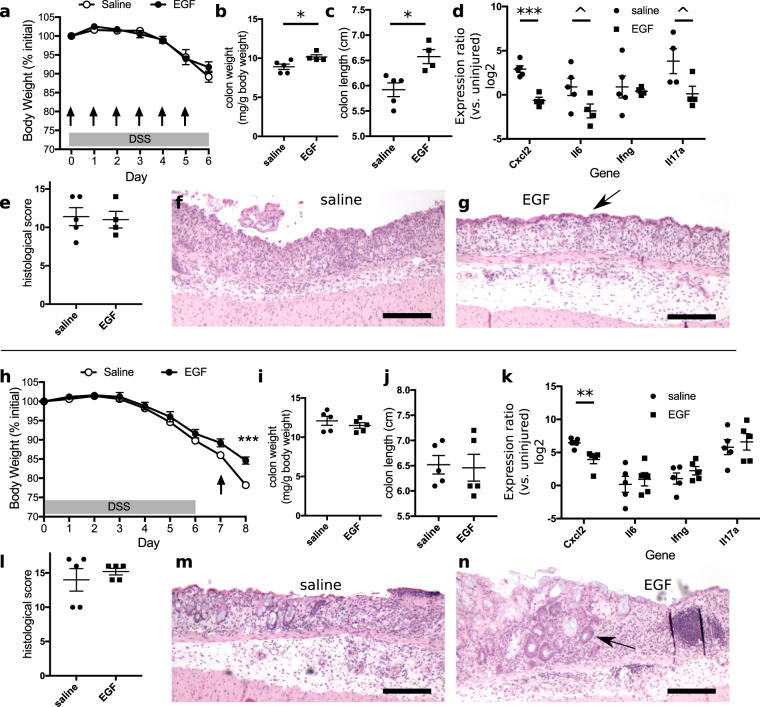
EGF treatment improves outcomes of DSS colitis. (**a**) Simultaneous oral DSS administration and daily intraperitoneal injection of 1 µg EGF did not alter body weight loss compared to saline-injected controls. The arrows indicate the days in which daily doses of saline or EGF were injected. (**b**,**c**) Both colonic weight (**b**) and length (**c**) were increased by EGF treatment, supporting trophic activity of EGF in the context of injury. (**d**) EGF injection for 6 d resulted in decreased expression of proinflammatory colonic cytokines *Cxcl2*, *Il6*, and *Il17a*. (**e**) There were no discernible differences in overall colonic histological score in mice treated with EGF or saline. (**f**,**g**) H&E-stained sections demonstrate that saline-treated samples (**f**) exhibited qualitatively greater ulceration than EGF-treated samples (**g)**. The arrow indicates surviving surface epithelial cells in EGF-treated samples. Images were acquired at 40× magnification. (**h**) In contrast to treatment with EGF during DSS exposure, intraperitoneal 1 µg EGF injection after DSS withdrawal limited further body weight loss. (**i**,**j**) No differences in colon weight (**i**) or colon length (**j**) were observed between EGF and saline-treated specimens. (**k**) The EGF-treated group exhibited decreased colonic *Cxcl2* expression on day 8. (**l**) Overall histological scores did not differ between treatment groups. (**m**,**n**) H&E-stained sections of colons from saline-treated animals demonstrated ulceration and crypt loss (**m**). Similar characteristics were observed in colons from EGF-treated mice (**n**). However, groups of regenerative crypt structures (arrow) were noted in all of the EGF-treated samples (**n**). Images were acquired at 40× magnification. ^p < 0.1; *p < 0.05; **p < 0.01; ***p < 0.001. Summary statistics: mean ± s.e.m. Scale bars: 200 µm.

While gefitinib induced profound exacerbations in colonic injury and inflammation, a more clinically relevant question was whether improvements in outcomes could be observed with treatment with EGF. We therefore tested in the DSS model if EGF administration ameliorated colonic injury and altered cytokine expression. In mice that received 1 µg/d EGF concomitant with DSS exposure (i.e., on a similar schedule as shown in Fig. 1a), body weight loss during DSS treatment was similar to that in DSS-treated mice receiving vehicle (n = 5 mice per condition, Fig. 3a). Surprisingly, colonic weights increased with EGF treatment (p = 0.03, Fig. 3b). However, these increases in weight were not accompanied by the shortening of the colon typically observed in severe injury. Instead, the colons of EGF-treated mice were longer than those of saline-treated controls (p = 0.01, Fig. 3c). Importantly, expression of *Cxcl2* was significantly reduced (by 91%, p = 0.0003), and transcript levels of *Il6* (reduction of 85%, p = 0.08) and *Il17a* (reduction of 92%, p = 0.07) but not *Ifng* (p = 0.71) trended downward (Fig. 3d). No changes were observed in overall histological colitis score due to EGF treatment (p = 0.99, Fig. 3e). A qualitative microscopic examination of the colitis suggested improved surface epithelialization in the colons of EGF-treated animals (Fig. 3f,g). Thus, while EGF could not prevent the onset of colonic injury, it improved the inflammatory profile and may ameliorate surface ulceration associated with the injury.

Intraperitoneal administration of EGF during the healing phase of the DSS injury model (i.e., on a similar schedule as shown in Fig. 2) resulted in dramatic improvement in body weights (Fig. 3h, n = 5 mice per condition). However, these improvements were not reflected as EGF-induced changes in colonic weights (Fig. 3i) or length (Fig. 3j). EGF significantly reduced expression of *Cxcl2* (reduction of 83%, p = 0.007), while no significant changes were detected in *Il6* (p = 0.64), *Ifng* (p = 0.30), and *Il17a* (p = 0.64) (Fig. 3k). No significant differences in colonic histological score were found between saline- and EGF-treated animals (p = 0.99, Fig. 3l), but all colons (5/5, 100%) in the EGF-treated group exhibited clusters of regenerative crypts at the boundaries of ulcerations. Regenerative changes were found in 3/5 (60%) colons in the saline-treated group (Fig. 3m,n).

### Onset time-dependent effects of EGFR modulation in colitis-associated tumorigenesis

**Figure 4:**
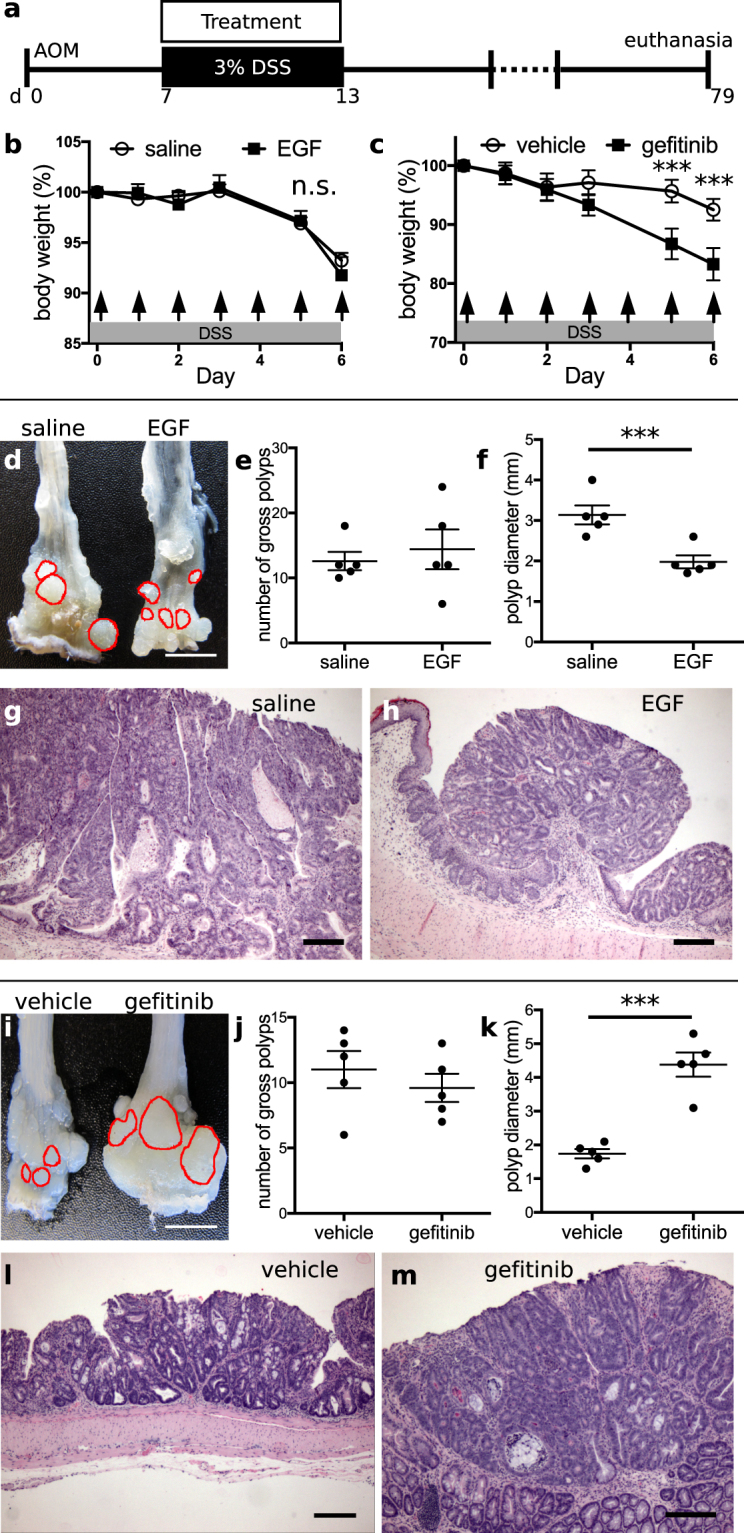
EGFR activation during active colonic injury reduces tumor size. (**a**) Animals were exposed to the AOM-DSS colitis-associated cancer model consisting of an intraperitoneal injection of 12.5 mg/kg azoxymethane (AOM) followed after 7 d by a single round of 3% DSS injury for 6 d. Treatments consisted of either 1 µg/d EGF or saline intraperitoneal injections, or oral gavage of either 200 mg/kg/d gefitinib or control diluent (vehicle), given during the DSS injury cycle. (**b**,**c**) Administration of EGF did not alter body weight loss (**b**), but treatment with gefitinib replicated the pattern of accelerated weight loss (**c**) shown in Fig. 1. Arrows indicate treatment days. (**d**) EGF treatment reduced tumor size, as shown by gross analysis of the distal colonic surface, with select polyps outlined in red. (**e**,**f**) Quantification of EGF’s effects showed no change in tumor number in the distal colon (**e**) but a significant reduction in tumor size (**f**). Each dot on the plot in (**f**) represents the mean tumor diameter observed from all tumors found in a single animal. (**g**,**h**) H&E-stained sections reveal the polypoid structures and size disparity of tumors obtained from saline- (**g**) and EGF-treated (**h**) mice. Images were acquired at 40× magnification. (**i**–**k**) Oral gavage of gefitinib similarly did not affect tumor number, but increased tumor size in the distal colon, as shown in gross images of the colonic mucosal surface (**i**). Polyps were manually identified, counted (**j**), and their diameters measured (**k**) and compared between treatment groups. (**l**,**m**) Histological sections demonstrate the overall structure of tumors and their size difference between vehicle (**l**) and gefitinib-treated animals (**m**). Images were acquired at 40× magnification. *p < 0.05; **p < 0.01; ***p < 0.001. Summary statistics: mean ± s.e.m. Scale bars: (**d**,**i**) 5 mm, (**g**,**h**,**l**,**m**) 200 µm.

**Figure 5:**
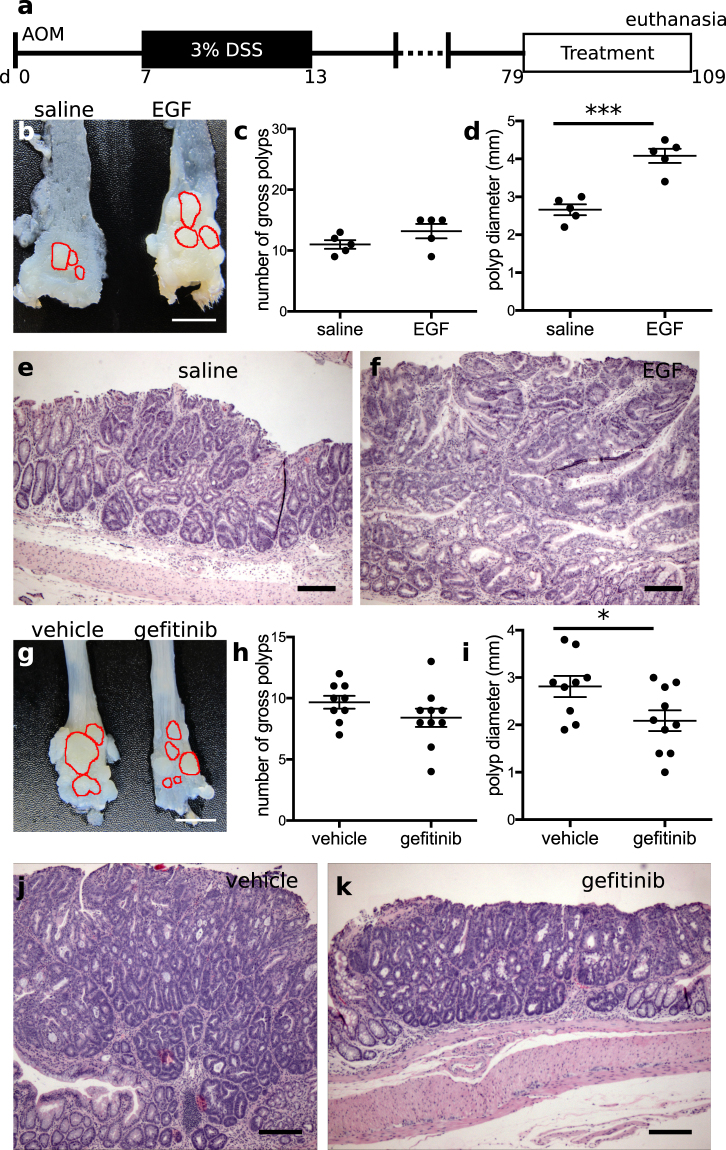
EGFR activation after mucosal healing and tumor initiation increases tumor size. (**a**) 1 µg EGF, saline, 200 mg/kg gefitinib, or vehicle were given to mice for 30 d, beginning 2 months after mutagenesis (AOM) and induction of colonic injury (DSS). (**b**–**d**) Gross images (**b**) of the distal colonic surface demonstrated that EGF increased tumor size, which is quantified by tumor multiplicity and diameter shown in (**c**) and (**d**), respectively. The perimeters of select polyps are outlined in red (**b**). (**e**,**f**) H&E-stained sections of distal colonic polyps from saline- (**e**) or EGF-treated (**f**) samples show the microstructures of highly dysplastic colonic epithelium which is enlarged in EGF-treated animals. Images were acquired at 40× magnification. (**g**–**i**) Photos (**g**) of anatomical grossing of gefitinib-treated colons in this experimental schedule showed unchanged tumor number (**h**) but decreased tumor size (**i**). (**j**,**k**) The larger size of vehicle-treated (**j**) versus gefitinib-treated (**k**) tumors is also apparent in H&E-stained sections of distal colonic mucosa. *p < 0.05; **p < 0.01; ***p < 0.001. Summary statistics: mean ± s.e.m. Scale bars: (**b**,**g**) 5 mm, (**e**,**f**,**j**,**k**) 200 µm.

EGFR modulation was observed to affect both epithelial cell proliferation and inflammation, key parameters that may regulate the aggressiveness of injury-associated tumors. To determine whether EGFR activation or inhibition could alter outcomes of colitis-associated cancer (CAC), we administered EGF or gefitinib to mice subjected to a modified azoxymethane/DSS CAC model. We modified the AOM-DSS model to reduce, from 3 to 1, the number of DSS cycles after AOM administration; this change allowed tumor initiation to occur within a single temporal window, instead of several windows corresponding to multiple later rounds of DSS treatment. In the modified AOM-DSS model, mice were injected with a single dose of AOM, and a 6 d course of 3% DSS was initiated 1 wk later. Outcomes of polyp number and size along the length of the colon were evaluated at either 65 d (experiment schedule 1, shown in Fig. 4) or 95 d (experiment schedule 2, shown in Fig. 5) after the cessation of DSS treatment. These two different schedules were designed to evaluate EGFR’s effects in a highly injurious/inflammatory state (schedule 1) or a healed state (schedule 2), potentially allowing differential assessment of EGFR’s tumor-altering functions in the presence or absence of injury. In the first set of experiments (experiment schedule 1, or “early phase” experiments), we treated mice daily with intraperitoneal EGF, oral gefitinib, or vehicle during DSS exposure (Fig. 4a, n = 5 mice per condition). In EGF-treated mice, there was no difference in weight loss during DSS administration (Fig. 4b). However, gefitinib-treated animals lost more weight than vehicle-treated controls (Fig. 4c). These weight curves replicate the results shown in Figs 1a and 3a. During anatomical examination (Fig. 4d), we found no difference in tumor number between EGF- and saline-treated animals (p = 0.61, Fig. 4e) but a 37% reduction (p = 0.003) in tumor diameter (Fig. 4f). In both experimental and control animals, the dysplastic tumor epithelium was contained within a polypoid structure emerging from a base layer of non-dysplastic epithelium (Fig. 4g,h). In gefitinib-treated mice (Fig. 4i–k), tumor number was unchanged (p = 0.45, Fig. 4j), but there was a 150% increase (p = 0.0001) in tumor size (Fig. 4k), which was also evident upon histologic examination (Fig. 4l,m). In all samples, the tumors were highly proliferative, exhibiting high concentrations of KI-67+ cells throughout the dysplastic glandular structures, and demonstrated that β-catenin (CTNNB1) was localized to the nucleus, where it can regulate transcription and support active WNT signaling. In contrast, KI-67+ cells in surrounding non-dysplastic areas of epithelium were primarily localized to the crypt base, and β-catenin protein appeared to be localized to the cell membrane, consistent with reduced WNT signaling (Supplementary Fig. S1). In total, these results indicate that activation of EGFR signaling during injury and inflammation improves tumor outcomes.

In the second series of experiments (experiment schedule 2, or “late phase” experiments) shown in Fig. 5, gefitinib, EGF, or vehicle was administered beginning at 65 d after the cessation of DSS, and continuing for ~1 mo until dissection (Fig. 5a, n = 5 mice per condition). No differences in DSS-induced weight loss were observed between groups (data not shown). Gross examination of specimens revealed no differences in colonic tumor number (p = 0.15 for EGF vs. saline in Fig. 5b–d, p = 0.19 for gefitinib vs. vehicle in Fig. 5g–i). However, tumor diameter increased by 53% (p = 0.0003) in EGF-treated mice and decreased by 26% (p = 0.03) in gefitinib-treated animals (Fig. 5b–g). This difference was also apparent in H&E-stained sections (Fig. 5e,f,j,k). Similar to the studies shown in Fig. 4 and Supplementary Fig. S1, polyps contained a highly proliferative (i.e., KI-67+) mass of cells and an abundance of nuclear-localized β-catenin (Supplementary Fig. S2). Thus, EGFR modulation had opposing effects on tumor burden when pharmacological agents were administered after mucosal healing (Fig. 5) compared to active injury (Fig. 4).

### Pharmacological EGFR modulation in the absence of injury

To understand how EGFR modulation affects colonic homeostasis, we administered EGF (intraperitoneal injection) or gefitinib (oral gavage) daily for 6 d to unchallenged (i.e., DSS-naive) mice (n = 5 mice per condition). The mice were then analyzed immediately at the end of the treatment. We reasoned that an understanding of how these agents affected colonic mucosa in the disease-free state might provide a mechanism for their activities in colitis and colitis-associated carcinogenesis. Moreover, an intriguing observation was EGF’s ability to decrease cytokine expression even in the absence of clear histological improvements in injury (Fig. 3d,k). We therefore also tested whether the effects of EGFR modulation on cytokine expression could still be observed in the absence of injury.

**Figure 6:**
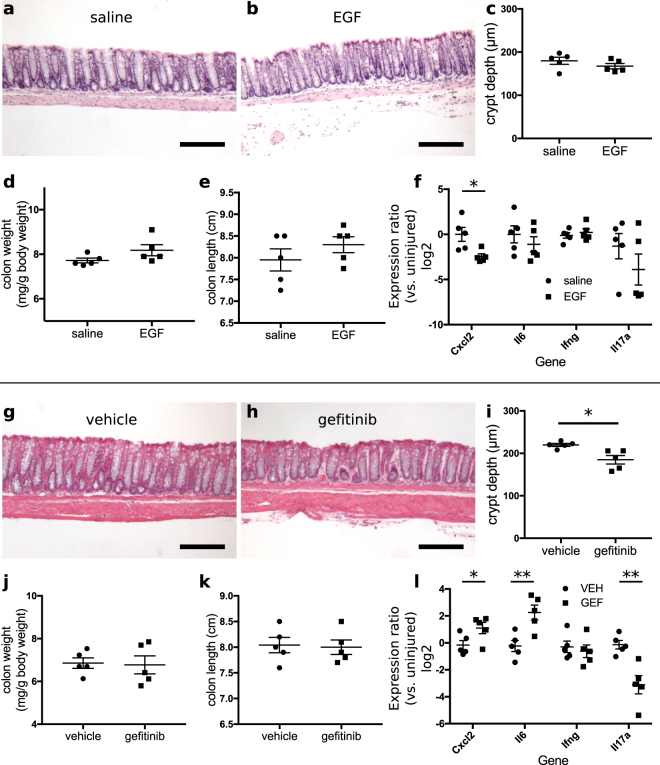
EGFR activity regulates colonic cytokine expression in the absence of injury. (**a**,**b**) Daily intraperitoneal 1 µg EGF injections for 6 d did not change overall structure of colonic crypts, as shown in H&E-stained sections of saline-treated (**a**) or EGF-treated (**b**) mice. (**c**–**e**) No changes due to EGF treatment were observed in average crypt depth (**c**), colon weight (**d**), or colon length (**e). **(**f**) EGF treatment resulted in decreased colonic expression of *Cxcl2*, but expression levels of *Il6*, *Ifng*, and *Il17a* were unchanged. (**g**–**i**) Daily gavage administration of 200 mg/kg gefitinib (GEF) for 6 d reduced average colonic crypt height, as shown in comparative H&E-stained sections of vehicle-treated (**g**) and gefitinib-treated (**h**) samples, with associated quantification (**i**). (**j**,**k**) This schedule of gefitinib administration did not impact colon weight (**j**) or colon length (**k). **(**l**) Administration of gefitinib (GEF) led to increased expression of *Cxcl2* and *Il6*, but decreased expression of *Il17a*. *p < 0.05; **p < 0.01; ***p < 0.001. Summary statistics: mean ± s.e.m. Scale bars: 200 µm.

No changes in body weight were observed to correlate with administration of EGF compared to saline (data not shown). Neither the overall crypt pattern (Fig. 6a,b), crypt depth (p = 0.26, Fig. 6c), colon weight (p = 0.13, Fig. 6d), nor colon length (p = 0.30, Fig. 6e) was affected by EGF. Intraperitoneal EGF administration significantly reduced *Cxcl2* mRNA expression (reduction of 31%, p = 0.03) and did not significantly alter *Il6* (p = 0.64), *Il17a* (p = 0.39), or *Ifng* (p = 0.54) expression (Fig. 6f). In contrast, administration of gefitinib did not change overall crypt shape (Fig. 6g,h) but resulted in crypt shortening (decrease of 16% in crypt height, p = 0.01, Fig. 6i). Overall colon weight (p = 0.86, Fig. 6j) and length (p = 0.85, Fig. 6k) remained unchanged. Treatment of animals with gefitinib increased the expression of *Cxcl2* (2.4 times, p = 0.04) and *Il6* (5.6 times, p = 0.007) but decreased expression of *Il17a* (reduction of 87%, p = 0.004) (Fig. 6l). Baseline *Ifng* levels were not affected by gefitinib treatment (p = 0.62). Thus, EGFR modulation induces subtle changes in colonic morphology and alters cytokine expression at baseline.

## Discussion

We have demonstrated that modulation of EGFR activity through administration of recombinant activating ligand (EGF) or small-molecule kinase inhibitor (gefitinib) alters outcomes of colonic injury, inflammation, and tumorigenesis. Administration of gefitinib during either the injury phase (Fig. 1a) or recovery phase (Fig. 2a) accelerated weight loss in the DSS-induced colonic injury model. The gefitinib-associated increases in colon weight (Fig. 1b) and reductions in colon length (Figs 1c, 2c) were consistent with exacerbated injury. Gefitinib had profound effects on epithelial cell function. In homeostasis, treatment of mice with gefitinib daily for 6 d induced shortening of the colonic crypt (Fig. 6i), possibly due to accelerated shedding of differentiated cells or reduced proliferation of the stem cell compartment, resulting in overall epithelial cell loss. The data suggest that reduced proliferation at the crypt base, where stem cells reside, is a strong candidate explanation. Genetic inhibition of EGFR signaling results in reduction of crypt cell proliferation^52^. In studies presented here, gefitinib restricted epithelial cell proliferation during DSS injury (Fig. 1d,e), which supports a critical role for EGFR in mediating the epithelial proliferative response to inflammation. With reduced EGFR signaling, colonic epithelium cannot proliferate to efficiently mediate re-epithelialization and crypt regeneration, especially in the recovery phase beginning after withdrawal of DSS. We believe these defects translate to worsened colonic outcomes in gefitinib-treated mice.

In contrast to deleterious outcomes of colitis associated with treatment with gefitinib, treatment of animals with EGF during DSS-induced injury improved overall outcomes. While EGF’s effects on body weight loss were overall modest (Fig. 3a,h), EGF had profound effects on pro-inflammatory cytokine expression. DSS exposure elevated the colonic expression of *Cxcl2*, *Ifng*, *Il6*, and *Il17a* (e.g., levels of expression in controls in Figs 1g, 2f and 3d,k are greater than 0 relative to uninjured samples). When administered during the injury phase, EGF abrogated the normal elevation of cytokines associated with DSS exposure (i.e., expression of cytokines in EGF-treated samples was at or near the 0-level relative to uninjured controls) (Fig. 3d). When given during the recovery phase, EGF reduced cytokine expression, but their levels remained higher than those in the colons of uninjured animals (Fig. 3k). Daily EGF treatment for 6 d was insufficient to induce crypt hyperplasia (Fig. 6a–e), supporting a high level of baseline EGFR activation in colonic epithelium. During injury, both colon weight and length were increased after EGF administration (Fig. 3b,c), suggesting that EGF may have some context-dependent trophic functions. However, the consistent modulation of cytokine expression by EGF and gefitinib supports that a major potential mechanism for EGF’s restriction of injury is the direct attenuation of proinflammatory cytokine expression. These effects were discernible even in unchallenged mice (Fig. 6f), but their physiological importance was likely amplified during colitis such that differences in overall outcome were significant. While the precision of the histological scoring system^63,64^ used to quantify colitis severity by accounting for combined immune infiltration, ulceration, and crypt remodeling may be insufficient to identify significant gefitinib- or EGF-induced changes (Figs 1f, 2e and 3e,l), differences in proinflammatory cytokine expression may underlie downstream changes in crypt loss or mucosal healing (as depicted in Figs 1e, 2d and 3f,g,m,n).

Of the four tested inflammatory genes, EGF or gefitinib consistently regulated *Cxcl2*, a key chemokine involved in the attraction of neutrophils to sites to injury. CXCL2 is secreted by macrophages and intestinal epithelium^78–80^. An attractive hypothesis is that EGFR signaling restricts epithelial CXCL2 expression, thereby limiting recruitment of neutrophils, key components of the immune infiltrate in colitis, after DSS-induced epithelial damage. These data suggest a complementary mechanism (i.e., via direct alteration in epithelial cytokine expression) through which EGFR signaling can improve outcomes of colitis, in addition to EGFR’s classically known roles in the promotion of epithelial cell proliferation, survival, and restitution. Future studies will need to define the roles of EGFR signaling in the regulation of epithelial innate immunity and to disentangle these effects from those in intestinal myeloid cells^58,81,82^.

A major concern with EGFR-activating therapies in IBD is that they will increase the burden of colitis-associated cancer. However, previous preclinical work utilizing mice with dominant-negative (inactivating) EGFR mutations paradoxically showed an increase in cancer incidence^52^. Using the AOM-DSS model here, which allowed for experimental separation of tumor initiation/early promotion from late promotion, we demonstrated that the protective effect of EGFR signaling is temporally governed. Tumors were smaller when EGF was given during injury/colitis (or early) phase (Fig. 4d–h), but larger when EGF was given during the healed/remission (or late) phase (Fig. 5b–f). The opposite results were obtained when EGFR kinase activity was inhibited with gefitinib (Figs 4i–m and 5g–k), supporting EGFR modulation as an important “switch” in determining tumor outcomes in a manner dependent on both time of intervention and the underlying disease state. These results suggest that pharmacological targeting of EGFR activation during active flares may be effective in reducing the burden of CAC, but may be deleterious once dysplastic lesions are present (e.g., if the patient has had a long history of under-treated disease). EGFR activation during the injury phase did not reduce the overall number of tumors, likely because mutational effects of AOM, and not differential colitis severity (Fig. 4b,c), in this model are the primary drivers of tumor initiation. We believe the reduction in tumor size associated with early EGF treatment was due to EGFR-induced changes in the tumor microenvironment, including decreased cytokine expression, resulting in early epigenetic, mutational, or clonal reprogramming of tumor cells that retarded their overall growth. For example, inflammation-associated adenomas may subvert proinflammatory signals, converting them from restrictive signals into proliferative signals that promote tumor outgrowth (as previously^83–85^ suggested). During an active flare, EGF’s anti-inflammatory effects may dominate over its activation of classically oncogenic pathways. In contrast, the late-stage effects of EGFR activation on tumor promotion likely stem from the direct activation of proliferative, migratory, and survival pathways in the tumor cells themselves.

These results are overall consistent with those obtained in clinical trials on the potential use of EGF enemas to treat UC^55^, and with preclinical data generated from experiments performed on genetically modified mice^52^. The preclinical data obtained here using pharmacological approaches to target EGFR warrant longer-term and larger-scale studies of EGFR activation to treat IBD. Of particular interest may be strategic formulations that allow for renewal of intellectual property. For example, small-molecule screening for novel EGFR activators may have value. In addition, utilization of genetically modified microbes (probiotics) and food crops to produce EGFR ligands on a large scale may provide cost-effective delivery of therapeutic quantities^86^. We have shown that specific proteins in probiotics activate colonic EGFR and restrict colitis^87^. Novel EGFR-targeting formulations produced at-scale will make longer-term evaluations of the oncogenic implications of EGFR-targeted therapies possible.

## Electronic supplementary material

Supplementary Figures
