# Supplementary material for: Pharmacological activation of epidermal growth factor receptor signaling inhibits colitis-associated cancer in mice
Source: Sci Rep. 2018 Jun 14;8:9119. doi: 10.1038/s41598-018-27353-w (PMC6002410; doi:10.1038/s41598-018-27353-w)
Supplement: Supplementary file 1 — Supplementary Figures [file 41598_2018_27353_MOESM1_ESM.pdf]

SUPPLEMENTARY FIGURES

Pharmacological activation of epidermal growth factor receptor signaling inhibits colitis-associated cancer in mice

Philip E. Dubé[1,2]*, Cambrian Y. Liu[1]*, Nandini Girish[1], M. Kay Washington[3], D. Brent Polk[1,4+]

* Authors contributing equally

[1] Division of Pediatric Gastroenterology, Hepatology, and Nutrition
Children's Hospital Los Angeles
Los Angeles, CA USA

[2] Present address: Taconic Biosciences
Hudson, NY USA

[3] Department of Pathology
Vanderbilt University Medical Center
Nashville, TN USA

[4] Department of Biochemistry and Molecular Medicine
Keck School of Medicine of University of Southern California
Los Angeles, CA USA

+ Correspondence: dbpolk@chla.usc.edu

Supplementary Figure S1

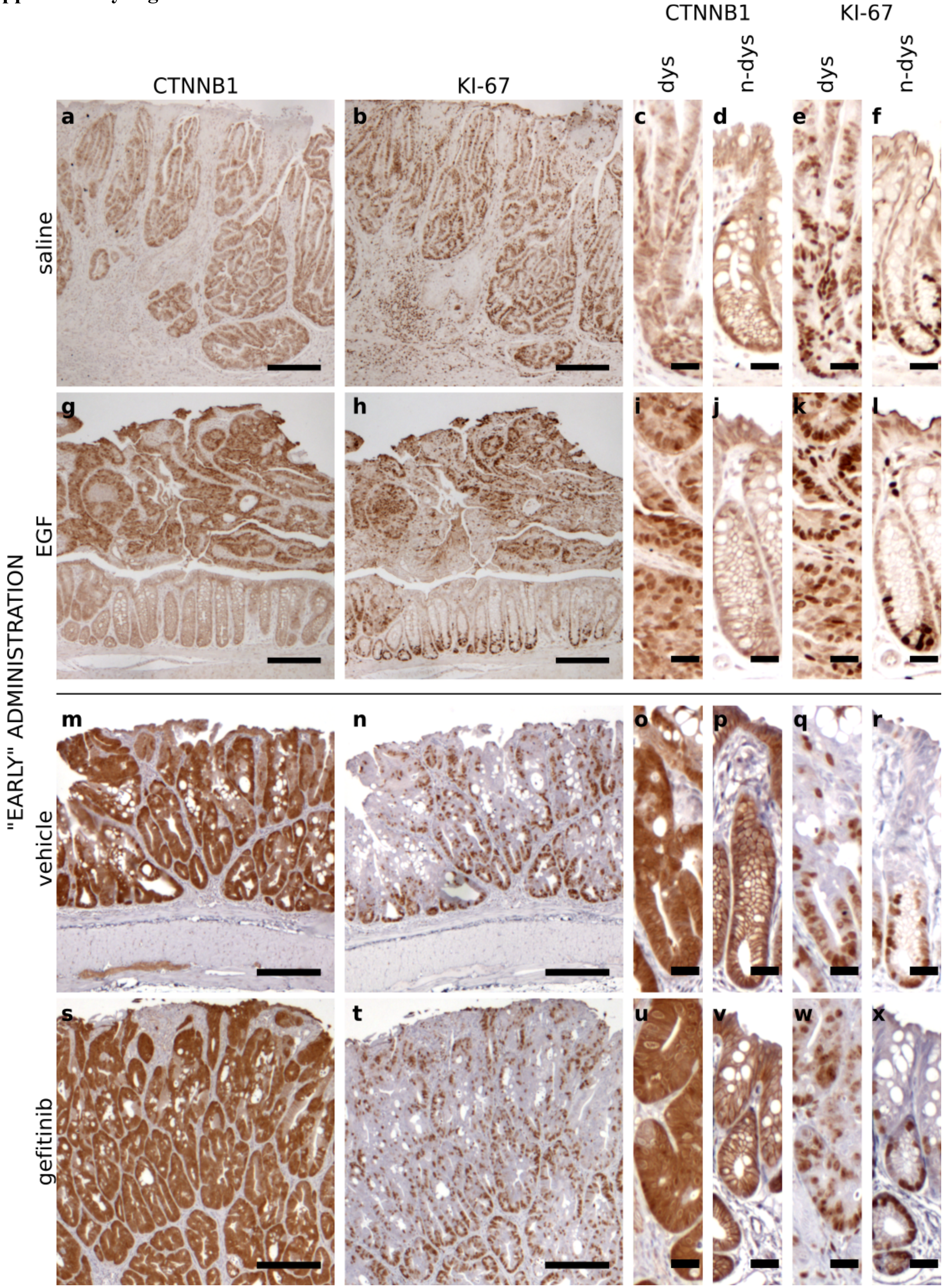

Supplementary Figure S1: β-catenin and KI-67 expression in colonic adenomas in mice treated with EGF, saline, gefitinib, or vehicle during DSS administration (i.e., "early" administration). Adjacent sections of colonic polyps ("dys" - dysplastic tissue) and matched normal mucosa ("n-dys" - non-dysplastic tissue) were stained with antibodies raised against the Wnt pathway component β-catenin (CTNNB1) or the cell proliferation marker KI-67. **a-f)** Saline-treated controls. **g-l)** EGF-treated samples. **m-r)** Vehicle-treated controls. **s-x)** Gefitinib-treated samples. Staining signal is brown with a blued hematoxylin counterstain. Low-magnification photos of dysplastic tissue are shown in (**a,b,g,h,m,n,s,t**). The other photos show high-magnification zooms. In all samples, β-catenin expression is enriched in epithelia, as background staining in the lamina propria is low. However, in dysplastic epithelial tissue the β-catenin signal is nuclear-localized, while in non-dysplastic epithelial tissue the signal is primarily membrane-localized. In all samples, KI-67 signal is in the nucleus. In dysplastic tissue, KI-67+ cells are found throughout the tissue, but in normal tissue, the positively stained cells are found in a restricted domain in the lower half of the colonic crypt. Scale bars: **a,b,g,h,m,n,s,t)** 200 μm; **c-f,i-l,o-r,u-x)** 30 μm.

Supplementary Figure S2

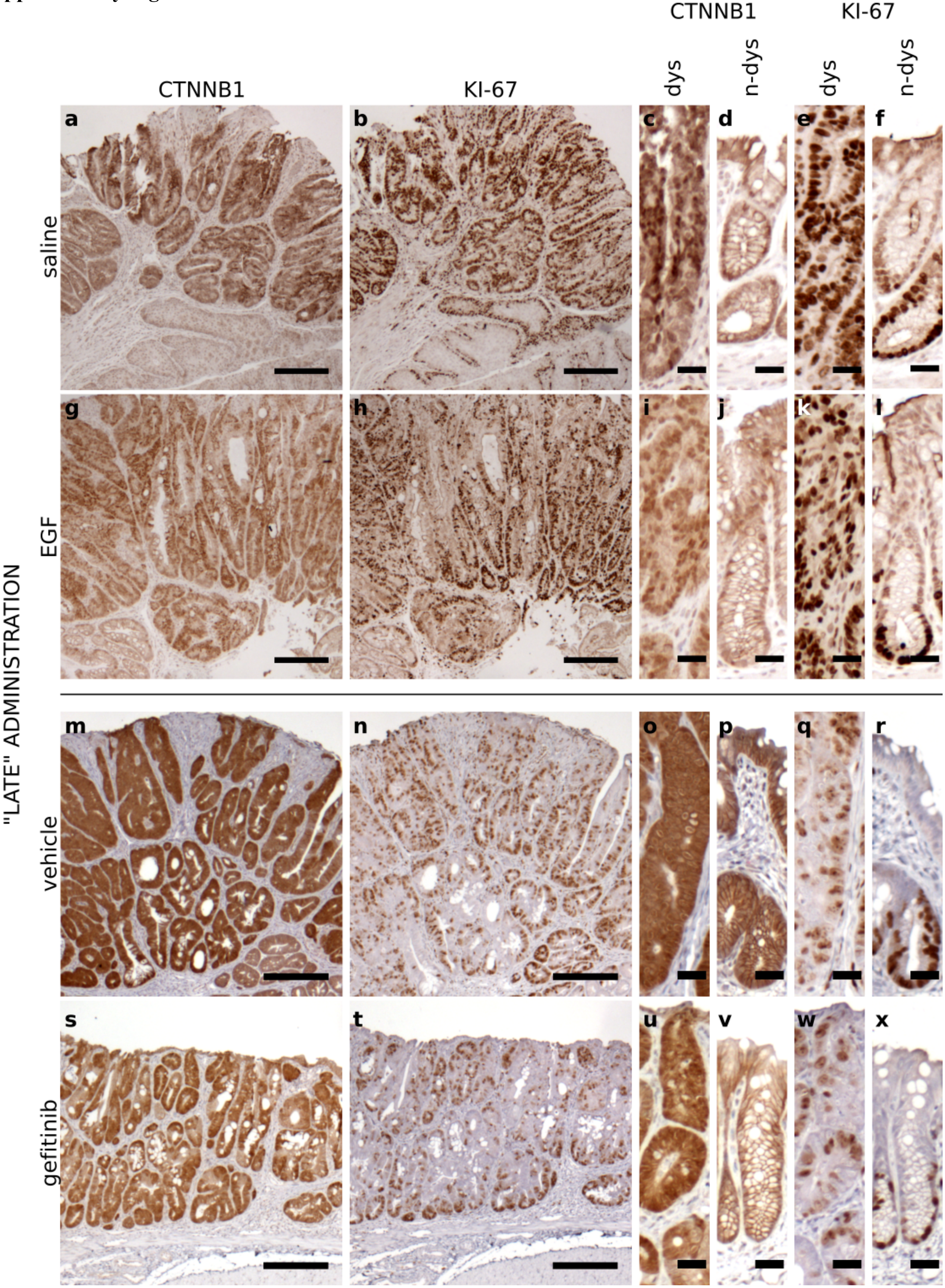

Supplementary Figure S2: β-catenin and KI-67 expression in colonic adenomas in mice treated with EGF, saline, gefitinib, or vehicle 2 months after DSS administration (i.e., "late" administration). Adjacent sections of colonic polyps ("dys" - dysplastic tissue) and matched normal mucosa ("n-dys" - non-dysplastic tissue) were stained with antibodies raised against β-catenin (CTNNB1) or the cell proliferation marker KI-67. **a-f)** Saline-treated controls. **g-l)** EGF-treated samples. **m-r)** Vehicle-treated controls. **s-x)** Gefitinib-treated samples. Staining signal is brown; a hematoxylin counterstain was also used to indicate tissue structure. Low-magnification photos of dysplastic tissue are shown in (**a,b,g,h,m,n,s,t**). The other photos show high-magnification zooms. Similar to Supplementary Fig. S1, β-catenin expression is primarily in colonic epithelium. However, in dysplastic tissue the β-catenin signal is nuclear-localized, but the signal is membrane-localized in normal tissue. In dysplastic tissue, KI-67+ cells are found throughout the tissue, from the base to the luminal surface of the polyp. In contrast, KI-67+ cells are only found near the crypt base in normal tissue. Combined with data shown in Supplementary Fig. S1, these results suggest that gefitinib and EGF do not change the fundamental nature of the colitis-associated tumors with respect to β-catenin signaling and that hyperproliferative tissue can be found in the tumors regardless of the treatment. Scale bars: **a,b,g,h,m,n,s,t)** 200 µm; **c-f,i-l,o-r,u-x)** 30 µm.
